# Comparative effects of physical education teaching models on motor skill development in school-aged children and adolescents: a systematic review and network meta-analysis

**DOI:** 10.3389/fpubh.2026.1905122

**Published:** 2026-10-06

**Authors:** Qi Tian, Yaowen Liu, Ziyu Wang, Gang Qin

**Affiliations:** 1 College of Physical Education, Chengdu University, Chengdu, China; 2 Department of Sports Science, Hanyang University, Seoul, Republic of Korea; 3 College of Sports, Sejong University, Seoul, Republic of Korea

**Keywords:** game-based learning, motor skill development, network meta-analysis, physical education, teaching models, TGfU

## Abstract

**Background:**

Motor skill development is a central objective of school physical education, yet the comparative effectiveness of teaching models remains unclear.

**Objective:**

This systematic review and network meta-analysis compared physical education teaching models for motor skill development in school-aged children and adolescents.

**Methods:**

Records were identified from Scopus (*n* = 300), Web of Science (*n* = 41), OpenAlex (*n* = 63), and PubMed (*n* = 33). After removal of 42 duplicates, 395 records were screened, 65 full texts were assessed, and 21 studies were retained in the systematic review. Thirteen studies contributed to the under-18 primary network restricted to school-based physical motor execution or sport-specific performance outcomes; 19 contributed to a broader sensitivity network. The protocol was registered in PROSPERO (CRD420261417609). Random-effects models estimated standardized mean differences (SMD) relative to Control and *P*-score rankings.

**Results:**

The primary network contained nine treatment nodes. Hybrid Teaching Games for Understanding–Sport Education (TGfU-SE), Nonlinear pedagogy, and Functional games showed the most favorable relative effect estimates in the primary network. However, the treatment ordering was sensitive to outcome definition, participant age, and individual studies, while substantial heterogeneity, network inconsistency, sparse comparisons, and risk of bias remained. In the broad sensitivity network, Cooperative had the highest *P*-score and estimated effect only while the single Akil visual-motor integration study was included; the node disappeared when that study was removed.

**Conclusion:**

Restricting the primary network to participants younger than 18 years improved population and outcome comparability and reduced heterogeneity, but significant inconsistency, sparse comparisons, and risk of bias preclude a definitive hierarchy. The *P*-score ordering should therefore be interpreted as a set of conditional effect estimates rather than proof that one teaching model is universally superior. In practice, physical education teachers should select and implement structured teaching models according to student age, learning objectives, sport content, and teaching context.

**Systematic review registration:**

https://www.crd.york.ac.uk/PROSPERO/view/CRD420261417609, identifier PROSPERO (CRD420261417609).

## Introduction

Motor skill development represents a foundational component of children’s physical, cognitive, and social growth. Proficiency in fundamental movement skills is widely treated as a critical prerequisite for lifelong physical activity participation and health promotion (1). A recent longitudinal systematic review and broader developmental synthesis confirmed that motor competence in childhood predicts later physical activity, health-related fitness, and weight status across the lifespan (1, 2). Research further suggests that children with higher motor competence are more likely to engage in physical activity during adolescence and adulthood, establishing a positive trajectory toward reduced risk of chronic diseases including obesity, cardiovascular disease, and type 2 diabetes (3). Beyond physical health benefits, motor skill proficiency has also been linked to cognitive, academic, and psychosocial development.

School-based physical education provides a unique and equitable platform to systematically cultivate motor competence across diverse student populations, as it reaches children regardless of socioeconomic background or access to extracurricular sports programs (4). International policy frameworks, including UNESCO’s Quality Physical Education guidelines, have emphasized the central role of physical education in developing motor competence as a foundation for lifelong physical literacy (5). Recent school-based syntheses have likewise shown that motor competence can be improved through deliberately designed instructional programs rather than being left to mature spontaneously (6).

Unlike interventions like community sports or extra-curricular activities which usually involve the participation of self-selecting members, physical education includes almost all children irrespective of their lack of previous experience or exposure to sports. This is why questions of efficacy of teaching models do not only concern teacher preference but also issues related to educational equality, physical literacy, and public health (7–9).

In the past two decades, physical education teaching methodology has moved from traditional teaching-centered methodologies, with some evidence-based instruction strategies emerging as popular ways of improving learning effectiveness (10). For instance, the Sport Education model is often mentioned due to its focus on authentic sporting practices, which are developed through long sporting seasons and student role assignment (11), and the Cooperative Learning model, which structures group interactions that result in improved physical and social skills acquisition (12).

Other prominent approaches include Teaching Games for Understanding (TGfU), which prioritizes tactical awareness and decision-making within game contexts (13), alongside inquiry-oriented or nonlinear approaches that position students as active problem-solvers in movement challenges (14). More explicit linear or technical approaches derived from direct instruction traditions also remain common in practice. These pedagogies differ not only in lesson format, but also in how they organize practice, feedback, peer interaction, task constraints, and decision-making opportunities. Meaningful differences in motor-related outcomes are therefore theoretically plausible even when the same sport content is taught.

It is clear from existing review articles that a structured or model approach is better than minimal guidance and routine learning. Nevertheless, the reviews do not address the much more applied issue concerning the identification of which models would be superior if compared to other models at the same time (15, 16). Moreover, an additional complication arises from the variety in the choice of outcomes for analysis – some studies consider gross motor skills, while others measure sports techniques and even performance on the pitch.

From a practical point of view, this unresolved comparative issue carries significance because, in practice, teachers do not make a decision between one approach and doing nothing. Instead, they make their decisions based on a range of other structured approaches under the limitation of time available per class, number of students, sequence of topics in curriculum, and evaluation processes. Although a particular framework may prove to be efficient for a limited set of circumstances, it does not necessarily provide the best solution when considering the limited information available, unexpected conceptual outcomes, or application in only one sport. Thus, comparative synthesis is necessary not only for determining suitable frameworks, but also for determining how reliable the ranking of models actually is (10, 16).

Network meta-analysis (NMA) offers a useful methodological response to this problem because it can synthesize both direct and indirect evidence across multiple teaching models within a connected network (17, 18). At the same time, rankings from a sparse educational network can be misleading if they are interpreted without attention to study count, outcome comparability, and inconsistency. The present review therefore aimed to compare the effects of multiple physical education teaching models on motor skill development in school-aged children and adolescents using NMA. More specifically, we sought to determine not only which nodes ranked higher than Control, but also which apparent ranking differences were supported by recurring evidence and which were driven by sparse or conceptually heterogeneous studies.

## Methods

### Protocol and reporting

This review was prepared in line with PRISMA 2020 guidance (19). The protocol for this systematic review and network meta-analysis was registered prospectively in the International Prospective Register of Systematic Reviews (PROSPERO; registration number: CRD420261417609). Because the present manuscript reports a network meta-analysis, we also interpreted the evidence according to core network meta-analytic principles, including attention to network connectivity, transitivity, consistency, and the uncertainty surrounding treatment ranking (17).

### Search strategy

A multi-database search was conducted using Scopus, Web of Science, OpenAlex, and PubMed. The final search set contained 437 records: 300 from Scopus, 41 from Web of Science, 63 from OpenAlex, and 33 from PubMed. Search terms combined physical education teaching models with motor-skill-related outcomes and school-age populations. Core concepts included “sport education model,” “cooperative learning,” “teaching games for understanding,” “game-based learning,” “nonlinear pedagogy,” “direct instruction,” “motor skill,” “motor competence,” and “physical education,” together with related variants. English- and Chinese-language studies were considered when sufficient quantitative information could be extracted.

### Eligibility criteria

Studies were eligible if they met the following criteria: (1) participants were school-aged children or adolescents enrolled in school settings; (2) the intervention was delivered within physical education or a closely related school-based instructional context; (3) the study compared at least one identifiable teaching model against a control condition or another teaching model; (4) the outcome included motor skill development, game performance, technical skill execution, or another clearly motor-related performance indicator; and (5) sufficient quantitative information was available to derive a standardized mean difference and place the comparison within a connected treatment network. We adopted a deliberately broad but still motor-oriented outcome definition because the physical education literature often reports motor competence through several neighboring constructs. Accordingly, eligible outcomes included gross motor skill batteries such as TGMD-derived measures (20), sport-specific technical skill tests, tactical-technical game-performance indicators, psychomotor composites, and balance or manipulative skill measures, provided that better scores reflected improved movement performance rather than solely motivation, enjoyment, or knowledge.

For the primary NMA, a stricter eligibility gate was applied: studies had to involve school-aged participants in school physical education and report physical motor execution, motor competence, or sport-specific motor performance. Each study also had to provide an independent between-pedagogy contrast. Studies outside the population, setting, or outcome gate were retained only in the broad sensitivity network when they still supplied a connected quantitative comparison. Risk-of-bias judgments were not used as an automatic exclusion criterion; studies satisfying the population, setting, comparison, outcome, data-availability, and connectivity requirements were retained, while bias judgments were addressed through visual coding, study-level sensitivity analyses, and cautious interpretation.

Studies were excluded from the systematic review if they were reviews, non-intervention studies, lacked a motor-related outcome or recognizable teaching model, or provided insufficient information for eligibility confirmation. Twenty-one studies were retained in the review. Yang C (40) and Mahedero (41) were described narratively but were not entered into either NMA because they did not provide an independent between-pedagogy comparison. The broad quantitative network therefore contained 19 studies. After applying the population, school-setting, physical-motor outcome, and age-younger-than-18 gates, the primary network contained 13 studies; Yang Q (42), which enrolled university men with a mean age of approximately 21 years, was retained only in the broad sensitivity network.

### Study selection

After removal of 42 duplicates, 395 unique records remained for screening. Title and abstract screening excluded 330 records. Sixty-five full texts were assessed for eligibility, and 44 were excluded for the following reasons: review articles (*n* = 18), non-intervention studies (*n* = 10), no motor skill measurement (*n* = 9), no teaching model intervention (*n* = 4), and other reasons (*n* = 3). Twenty-one studies were retained in the systematic review. This staged reduction was important because many initially relevant papers discussed pedagogical models in physical education but did not provide extractable motor-performance outcomes or a comparison structure that could contribute to a connected quantitative network.

For the present synthesis, an additional harmonization step was required after basic eligibility assessment. Some candidate studies reported multiple outcomes, hybrid interventions, or comparator labels that could not be entered directly into a small educational network without fragmenting the evidence base. We therefore selected one primary motor-related comparison per study and mapped intervention labels onto broader pedagogical nodes when the underlying teaching logic was substantively similar. This step was intended to preserve interpretability and network connectivity rather than to erase meaningful pedagogical nuance. The study selection process is summarized in Figure 1.

**Figure 1:**
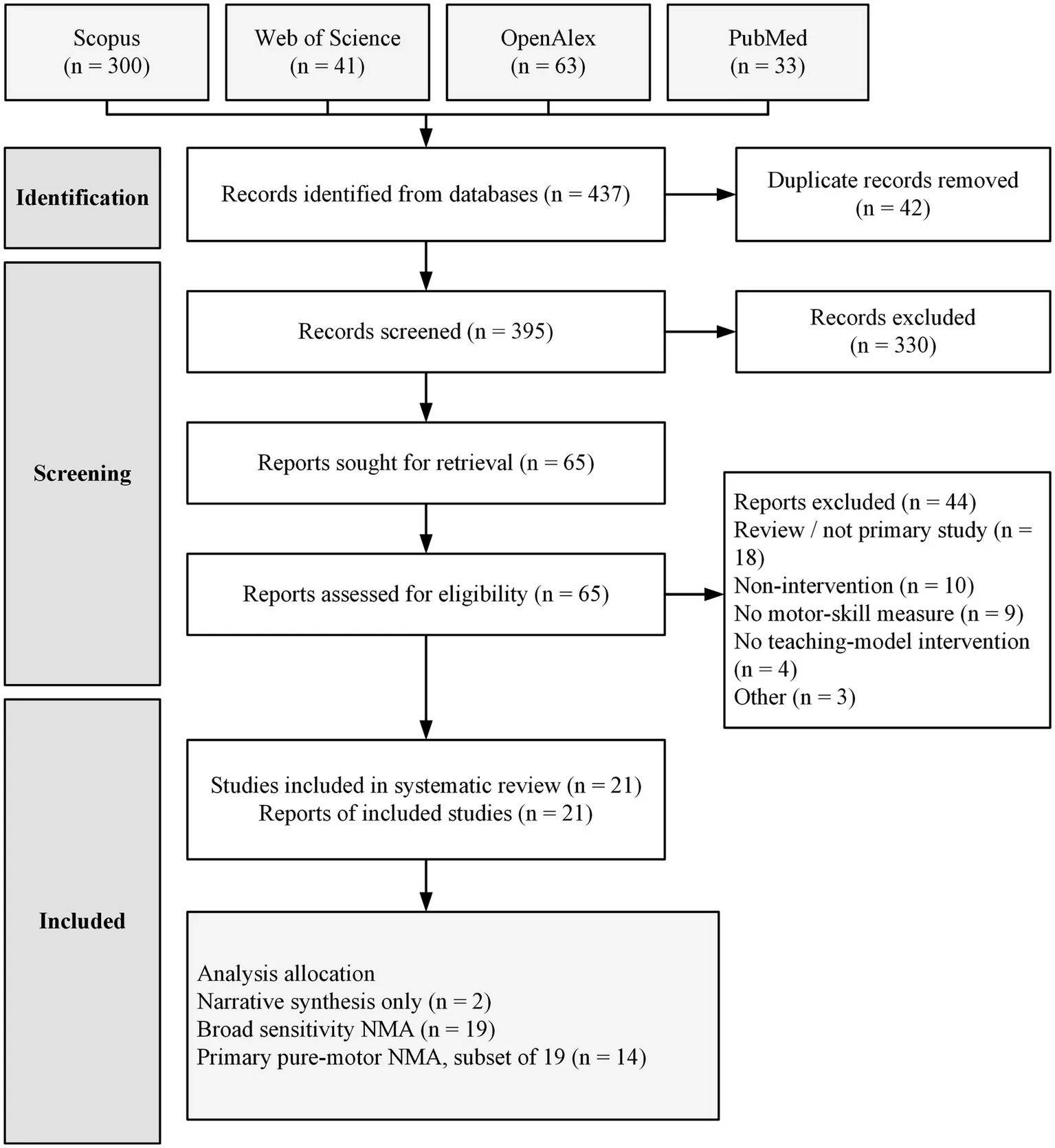
PRISMA 2020 flow diagram of study selection and analysis allocation.

### Data extraction and node harmonization

Data extraction covered study identifier, publication year, intervention and comparator, sample sizes, outcome type, arm-level summary statistics, effect estimate, variance source, and analytic role. The primary network comprised nine nodes: Control, Functional games, Game-based, Hybrid TGfU-SE, Linear, Nonlinear, Sport Education, TGfU, and Traditional. Cooperative appeared only in the broad sensitivity network because its sole contributing study, Akil (39), reported visual-motor integration rather than physical motor execution. Control denotes minimal or regular physical education comparison conditions, whereas Traditional denotes structured teacher-centered, technical, or classical instruction.

Study-level labels were mapped according to instructional logic rather than name alone. “Classical,” “Technical,” “Direct Instruction,” and related teacher-led comparators were assigned to Traditional or Linear according to the degree of explicit technical sequencing. “NLP” and related constraint-led or inquiry-oriented approaches were assigned to Nonlinear. Hybrid TGfU-SE and Functional games were retained as distinct nodes because their intervention structures were identifiable and connected. Six studies were reserved for the broad sensitivity network: Septiyanto (43) reported a mixed cognitive-psychomotor score; Akil (39) assessed visual-motor integration; Pugliese (44) supplied a cognitive-visual processing outcome; Práxedes (45) (45) involved youth club training; and Yang Q (42) and Espoz-Lazo (46) involved university participants. The operational definitions, study-level label examples, and analytic roles used for node assignment are summarized in Table 1. The revised under-18 primary network contains nine nodes; Cooperative is retained only in the broad sensitivity network (Table 2).

**Table 1:** Node harmonization criteria and analytic roles.

Harmonized node	Operational definition	Study-level labels mapped to node	Analytic role
TGfU	Explicit Teaching Games for Understanding or Tactical Games Model intervention.	TGfU; TGM; Tactical Games Model	Primary and broad networks
Hybrid TGfU-SE	Intervention explicitly combining TGfU and Sport Education components.	Hybrid TGfU-SE; TGfU-Sport Education hybrid	Primary and broad; kept separate from TGfU
Game-based	Comprehensive or tactical game-based model not explicitly identified as TGfU.	Game-based model; comprehensive model	Primary and broad networks
Functional games	Functional or developmental sports-game program structured around motor tasks.	Functional games; developmental or functional sports games	Primary and broad; Chinese school studies
Nonlinear	Nonlinear pedagogy or constraints-led manipulation of the task or environment.	Nonlinear pedagogy; NLP; MNL; constraints-led approach	Primary and broad networks
Sport Education	Standalone Sport Education model without explicit TGfU hybridization.	Sport Education; SEM	Primary and broad networks
Cooperative	Explicit cooperative-learning model.	Cooperative learning; cooperative model	Broad sensitivity only; Akil (39)
Traditional	Structured teacher-centered technical or skill-based instruction without explicit linear-pedagogy labeling.	Traditional; technical; skill-based; classical; direct instruction	Primary and broad; active comparator
Linear	Explicit linear pedagogy or linearly sequenced technical practice contrasted with a nonlinear approach.	Linear pedagogy; linear practice	Primary and broad; active comparator
Control	Usual PE, regular PE, or no specifically enriched instructional model.	Control; usual PE; regular PE	Primary and broad; reference node

**Table 2:** Characteristics of the included comparisons.

**Study**	**Country**	**Design**	**Comparison**	***N***	**Main motor-related outcome**
Septiyanto (43)	Indonesia	quasi-exp	TGfU vs. Control	67	cognitive and psychomotor tests
Práxedes (45)	Spain	quasi-exp	Nonlinear vs. Control	19	Pass skill execution
Portillo (49)	Spain	quasi-exp	Game-based vs. Traditional	47	game performance (good passes/catches/decisions)
Yang Q (42)	China	RCT	Nonlinear vs. Linear	40	Passing success rate; Variational Pass Rate; Variational Pass Success Rate
Nathan (50)	Malaysia	RCT	TGfU vs. Traditional	32	movement to base; decision-making; skill execution (in doubles game)
Akil (39)	Turkey	quasi-exp	Cooperative vs. Traditional	60	Bender-Gestalt Test (VMI); d2 Test of Attention
Pugliese (44)	Italy	quasi-exp	Nonlinear vs. Traditional	165	TGMD-3 (motor skills); Witty SEM (cognitive: divided attention, visual processing)
Sgro (48)	Italy	Quasi-experimental	TGfU vs. Traditional	67	GPAI Skill Execution Index
EslamiZad (51)	Iran	quasi-exp	TGfU vs. Linear	30	TGMD-2 (gross motor development); executive functions questionnaire
Arantes (52)	Brazil	quasi-exp	TGfU vs. Traditional	67	tactical-technical variables; life skills questionnaire
Espoz-Lazo (46)	Iran	quasi-exp	Nonlinear vs. Linear	23	Handball ability test
Tang (53)	China	quasi-exp	Sport Education vs. Traditional	116	table tennis skill tests (flat serve, backhand push-drive, forehand attack, half-table)
Invernizzi (54)	Italy	RCT	Nonlinear vs. Control	183	DMA (Dynamic Motor Creativity); executive functions (RNG task); self-efficacy; enjoyment
López-Lemus (55)	Spain	quasi-exp	Hybrid TGfU-SE vs. Traditional	137	Pass skill-execution index (GPAI)
Orangi (47)	Turkey	RCT	Nonlinear vs. Linear	64	Futsal shooting skill by motor-competence stratum
Nazari (56)	Iran	quasi-exp	Hybrid TGfU-SE vs. Control	72	Football motor skill
Yang C (40)	China	quasi-exp	Game-based vs. Traditional	59	Basketball Action Skills Test; ARCS Motivation Scale
Mahedero (41)	Spain	quasi-exp	Game-based vs. Traditional	33	game performance (decision-making, execution, game involvement)
ZhangYuhui (57)	China	quasi-exp	Functional games vs. Traditional	135	Fundamental motor skills: locomotor domain
LiuLongchang (58)	China	quasi-exp	Functional games vs. Control	73	Side-slip running time
ShenPeng (59)	China	quasi-exp	Functional games vs. Traditional	60	V-pattern layup time

A retrospective study-level risk-of-bias appraisal was undertaken after node harmonization. Comparisons described as randomized were appraised with RoB 2, whereas quasi-experimental or otherwise non-randomized school comparisons were appraised with ROBINS-I (21, 22). Because several reports provided only partial methodological detail or limited statistical tables, judgments were intentionally conservative and summarized at the overall study level in Table 3. For the primary network plot, each direct-comparison edge was assigned the highest overall risk-of-bias judgment among the studies informing that comparison. Red edges indicate at least one serious-risk study, whereas amber edges indicate comparisons informed only by studies rated as having some concerns. Edge width and the adjacent number represent the number of direct studies, and node size represents the total number of participants contributing to that treatment.

**Table 3:** Study-level risk-of-bias judgments.

Study	Tool	Overall judgment	Main reason for judgment
Septiyanto (43)	quasi-exp / ROBINS-I	Serious	Likely non-random class allocation; baseline comparability and outcome handling insufficiently reported.
Práxedes (45)	quasi-exp / ROBINS-I	Serious	Small non-randomized groups; confounding and selective outcome choice could not be ruled out.
Portillo (49)	quasi-exp / ROBINS-I	Serious	Quasi-experimental school allocation; blinding and baseline equivalence were unclear.
Yang Q (42)	RCT / RoB 2	Some concerns	Randomization was reported, but allocation concealment and assessor blinding were unclear.
Nathan (50)	RCT / RoB 2	Some concerns	Randomized comparison reported, but outcome handling and masking were insufficiently described.
Akil (39)	quasi-exp / ROBINS-I	Serious	Non-randomized comparison; confounding and assessor blinding were unclear.
Pugliese (44)	quasi-exp / ROBINS-I	Serious	Non-randomized comparison with incomplete variance reporting and selective-outcome concerns.
Sgro (48)	quasi-exp / ROBINS-I	Serious	Full-text quasi-experimental non-equivalent control-group design with intact classes; residual confounding and selection concerns remained after purposive/convenience sampling and exclusions from analysis.
EslamiZad (51)	quasi-exp / ROBINS-I	Serious	Non-randomized groups; methods and outcome tables were incompletely reported.
Arantes (52)	quasi-exp / ROBINS-I	Serious	Quasi-experimental design with limited detail on allocation and baseline equivalence.
Espoz-Lazo (46)	quasi-exp / ROBINS-I	Serious	Non-randomized comparison and incomplete quantitative reporting.
Tang (53)	quasi-exp / ROBINS-I	Serious	School-based non-randomized comparison with incomplete outcome-statistic reporting.
Invernizzi (54)	RCT / RoB 2	Some concerns	Randomized multi-arm study, but outcome reporting and masking details were limited.
López-Lemus (55)	quasi-exp / ROBINS-I	Serious	Non-randomized hybrid-unit comparison; baseline equivalence and blinding were unclear.
Orangi (47)	RCT / RoB 2	Some concerns	Randomization was reported, but concealment and subgroup outcome handling were insufficiently described.
Nazari (56)	quasi-exp / ROBINS-I	Serious	Quasi-experimental school design; potential confounding and selective reporting remained concerns.
Yang C (40)	quasi-exp / ROBINS-I	Serious	Non-randomized grouping comparison with limited methodological reporting.
Mahedero (41)	quasi-exp / ROBINS-I	Serious	Comparison within pedagogical contexts; incomplete reporting and likely confounding.
ZhangYuhui (57)	non-randomized / ROBINS-I	Serious	Incomplete report-level methods detail; confounding and reporting bias could not be ruled out.
LiuLongchang (58)	non-randomized / ROBINS-I	Serious	Incomplete report-level methods detail; allocation and outcome handling were unclear.
ShenPeng (59)	non-randomized / ROBINS-I	Serious	Incomplete report-level methods detail; confounding and reporting bias could not be ruled out.

### Statistical analysis

The 13-study under-18 primary NMA and the 19-study broad sensitivity NMA were conducted in R using the netmeta package under random-effects models estimated by restricted maximum likelihood. Standardized mean differences (SMD) were used as the common effect metric, with positive values favoring the intervention node over Control. A random-effects approach was chosen because substantial between-study variability was expected across age groups, sports, intervention formats, and outcome instruments. P-scores were used to order relative effect estimates; they do not quantify certainty of evidence. We therefore interpreted the P-score ordering alongside the number of contributing studies, the precision of confidence intervals, risk of bias, and network coherence rather than as stand-alone proof of superiority (17).

Inconsistency was assessed globally using the design-by-treatment decomposition and locally by separating the direct and indirect random-effects estimates for each comparison informed by both sources of evidence (node splitting). A two-sided *p*-value below 0.05 for the direct–indirect difference was considered evidence of local inconsistency. Heterogeneity was summarized with tau-squared and I-squared. Because the broad network showed significant global inconsistency, deletion diagnostics were also conducted for Septiyanto (43), Pugliese (44), and Akil (39), the three studies excluded from the nested physical-motor outcome network. All source articles were revisited for effect and variance data. In the 13-study under-18 primary network, 12 effects (92.31%) were calculated from reported arm-level sample sizes, means, and standard deviations; only Orangi (47) used reported competence-stratified Cohen’s d values and actual group sizes. In the 19-study broad sensitivity network, 17 effects (89.47%) used arm-level statistics, while Orangi (47) and Pugliese (44) used reported standardized effects and actual group sizes. No common, fixed, or otherwise assumption-based standard error was imputed. Established conversion principles were followed where reconstruction was required (23). The 13-study network was treated as the primary inferential analysis, whereas the 19-study network was reported as a broad sensitivity analysis. Rankings were interpreted conditionally because residual heterogeneity, inconsistency, and sparse direct comparisons remained. To examine whether the serious-risk Sgro (48) comparison materially affected the primary network, a study-specific deletion analysis was also performed after removal of that study.

To isolate the influence of outcome definition from the population and setting restrictions used in the primary analysis, we conducted a nested outcome-domain sensitivity analysis. The 19-study broad network was refitted after excluding Septiyanto (43), Akil (39), and Pugliese (44), whose outcomes were mixed cognitive-psychomotor, visual-motor integration, or cognitive-visual processing measures. This produced a 16-study physical-motor outcome network that retained Práxedes (45) and Espoz-Lazo (46), thereby changing outcome eligibility while holding the broader population and setting gate constant. For an exploratory subgroup assessment, studies were also classified as general/fundamental motor skill, sport-specific physical performance, or mixed/cognitive-perceptual outcomes. Network connectivity and closed loops were audited before subgroup rankings were considered; separate rankings were not reported for disconnected networks or connected trees without an independent cycle.

For the age analysis, studies were classified as child/preadolescent (≤12 years), adolescent (13–17 years), or young adult (≥18 years) using the source-reported age range, mean age, school grade, or age category. We refitted the broad network after excluding both young-adult studies and designated the 13-study under-18 physical-motor network as the primary analysis. Exploratory child/preadolescent and adolescent subgroup networks were fitted separately. Network connectivity and independent cycles were audited before subgroup rankings were interpreted; results from disconnected networks or connected trees without a closed loop were treated as descriptive estimability checks rather than definitive hierarchies.

A comparison adjusted funnel plot was generated to aid in the interpretation of small study effects using a descriptive approach. Due to the lack of density in the network, some nodes being based on a very small number of studies or even a single study, and because of differences in how outcomes were defined, publication bias was considered an exploratory analysis rather than a confirmatory one. In light of this, care was taken to draw the line between numerical ranking and evidence quality: a high *P*-score based on a single outlying comparison was not compared to a high moderate *P*-score based on several comparisons.

In light of the modest scale of the network under consideration, as well as multi-arm/hybrid designs in many of the included studies, methodological considerations pointed to prioritizing network consistency. Multi-arm studies were reduced to the contrast that was more consistent with the network nodes as established, while studies that presented contrasts that were either ambiguously defined or not strongly connected conceptually were kept from being influential. It is thus appropriate to understand this network as an analytically meaningful network of educational interventions, as opposed to every label in the literature.

## Results

### Study selection and included evidence

The search identified 437 records across four databases. After de-duplication and eligibility screening, 21 studies were retained in the systematic review. Yang C (40) and Mahedero (41) did not provide independent between-pedagogy contrasts and were therefore retained only in the narrative evidence table. Nineteen studies formed the broad sensitivity network. Thirteen studies met the primary-analysis gate for participants younger than 18 years in school-based physical education with physical motor execution or sport-specific performance outcomes.

The under-18 primary network contained nine nodes: Control, Functional games, Game-based, Hybrid TGfU-SE, Linear, Nonlinear, Sport Education, TGfU, and Traditional. Six studies were restricted to the broad sensitivity network because of mixed cognitive-psychomotor outcomes, visual or cognitive processing outcomes, university participants, or club rather than school physical education settings. This restriction reduced population and outcome differences in the effect-modifier distribution but did not eliminate heterogeneity or inconsistency.

The structure of the body of evidence also helps to explain the reason why some nodes are more stable than others. While the Game-based and TGfU approach reappear frequently among several different sports and age categories, the Cooperative and Sport Education approaches rely on significantly fewer connections. The nonlinear body of evidence is also diverse from a conceptual perspective, including not only the works examining game performance or creativity, but also the study by Pugliese (44), which compares the effects of a Nonlinear versus Traditional approach on an Italian school population, where the results include both TGMD-3 measures of motor performance and cognitive measures, thus placing it close to the boundary of the review’s definition of its outcomes. Practically, this means that nodes that consistently rank mid-range can provide more statistical power than those ranked at the top of the list based on a single comparison.

This network geometry matters for interpretation. Nodes linked repeatedly to Control or Traditional contribute more stable indirect contrasts across the network than nodes entering the model through a single comparison. As a result, the present network is more informative about whether active, structured approaches outperform minimal or conventional comparators than about fine-grained ordering among all active pedagogies.

The retrospective study-level risk-of-bias appraisal found that none of the four randomized comparisons was at low risk: all were rated as having some concerns under RoB 2, mainly because allocation concealment, masking, or outcome handling was insufficiently described. All 17 non-randomized comparisons were rated as having serious risk under ROBINS-I, primarily because of confounding, non-random allocation, or incomplete reporting of baseline equivalence and outcome handling. After the full report of Sgro (48) became available, its previous critical judgment based on abstract-only information was replaced by serious risk. The full text describes a quasi-experimental non-equivalent control-group design with intact classes, purposive and convenience sampling, and exclusions before analysis; these features leave substantial residual confounding and selection concerns but no longer justify a critical rating based solely on unavailable methods.

The 13-study under-18 primary network is presented in Figure 2. Ten studies were rated as having serious risk of bias and three as having some concerns. Of the 10 direct-comparison edges, eight included at least one serious-risk study and are shown in red; the remaining two were informed only by studies rated as having some concerns and are shown in amber. No direct comparison was supported exclusively by studies at low risk of bias.

**Figure 2:**
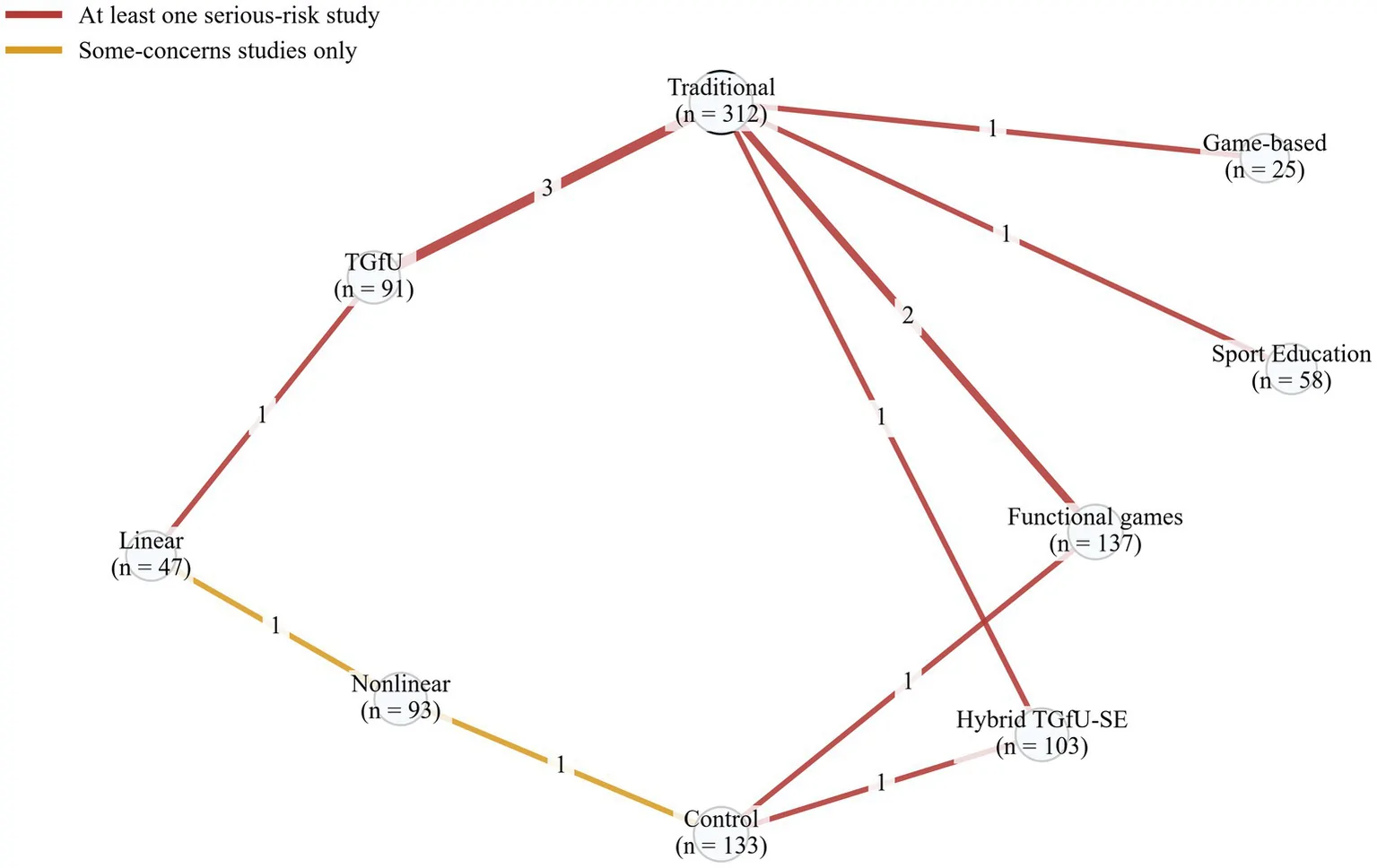
Risk-of-bias-coded network plot of the 13-study under-18 primary analysis. Node labels report the total number of participants contributing to each treatment; red edges indicate comparisons containing at least one serious-risk study, and amber edges indicate comparisons informed only by some-concerns studies. Edge width and labels represent the number of direct studies.

### Network meta-analysis findings

The 13-study under-18 primary network contained nine treatment nodes. Relative to Control, Hybrid TGfU-SE, Nonlinear, and Functional games had positive estimates with 95% confidence intervals that excluded zero. Estimates for Game-based, Sport Education, TGfU, Traditional, and Linear were imprecise and their confidence intervals crossed zero. Table 4 reports the conditional P-score ordering and effect estimates relative to Control, not a certainty-of-evidence hierarchy.

**Table 4:** Under-18 primary-network *P*-score ordering, study support, and effect estimates versus control.

***P*-score order**	**Teaching model**	**No. of studies**	***P*-score**	**SMD vs Control**	**95% CI**
1	Hybrid TGfU-SE	2	0.871	1.35	0.60 to 2.11
2	Nonlinear	2	0.812	1.24	0.47 to 2.01
3	Functional games	3	0.738	1.05	0.32 to 1.78
4	Game-based	1	0.670	0.90	−0.34 to 2.14
5	Sport Education	1	0.603	0.75	−0.40 to 1.89
6	TGfU	4	0.327	0.11	−0.76 to 0.97
7	Control	3	0.275	Reference	Reference
8	Traditional	8	0.189	−0.15	−0.90 to 0.61
9	Linear	2	0.013	−0.93	−1.92 to 0.06

“No. of studies” is the number of studies in the 13-study under-18 primary network that included each node; each study contributes to both nodes in its direct comparison. The count indicates replication within the network and is not a certainty-of-evidence rating.

The primary-network *P*-score ordering and estimated effects versus Control are presented in Table 4. *P*-scores summarize relative effects within the fitted network and do not represent certainty of evidence.

Hybrid TGfU-SE had the highest *P*-score in the under-18 primary network (*P*-score = 0.871; SMD = 1.35, 95% CI 0.60 to 2.11). This relative effect estimate was informed by two studies and was positive relative to Control, but its position in the P-score ordering should remain conditional because the network was sparse, heterogeneous, and inconsistent. The *P*-score does not constitute a certainty-of-evidence rating.

Nonlinear ranked second (*P*-score = 0.812; SMD = 1.24, 95% CI 0.47 to 2.01), and Functional games ranked third (*P*-score = 0.738; SMD = 1.05, 95% CI 0.32 to 1.78). Game-based and Sport Education had favorable point estimates but wide confidence intervals crossing zero. TGfU was also imprecise relative to Control (SMD = 0.11, 95% CI –0.76 to 0.97).

Traditional was approximately null relative to Control (SMD = –0.01, 95% CI –0.95 to 0.93), while Linear had a negative point estimate with substantial uncertainty (SMD = –0.45, 95% CI –1.57 to 0.66). The primary analysis therefore does not support the earlier claim that every active or structured teaching node outperformed Control.

The primary-network estimates relative to Control are displayed in Figure 3.

**Figure 3:**
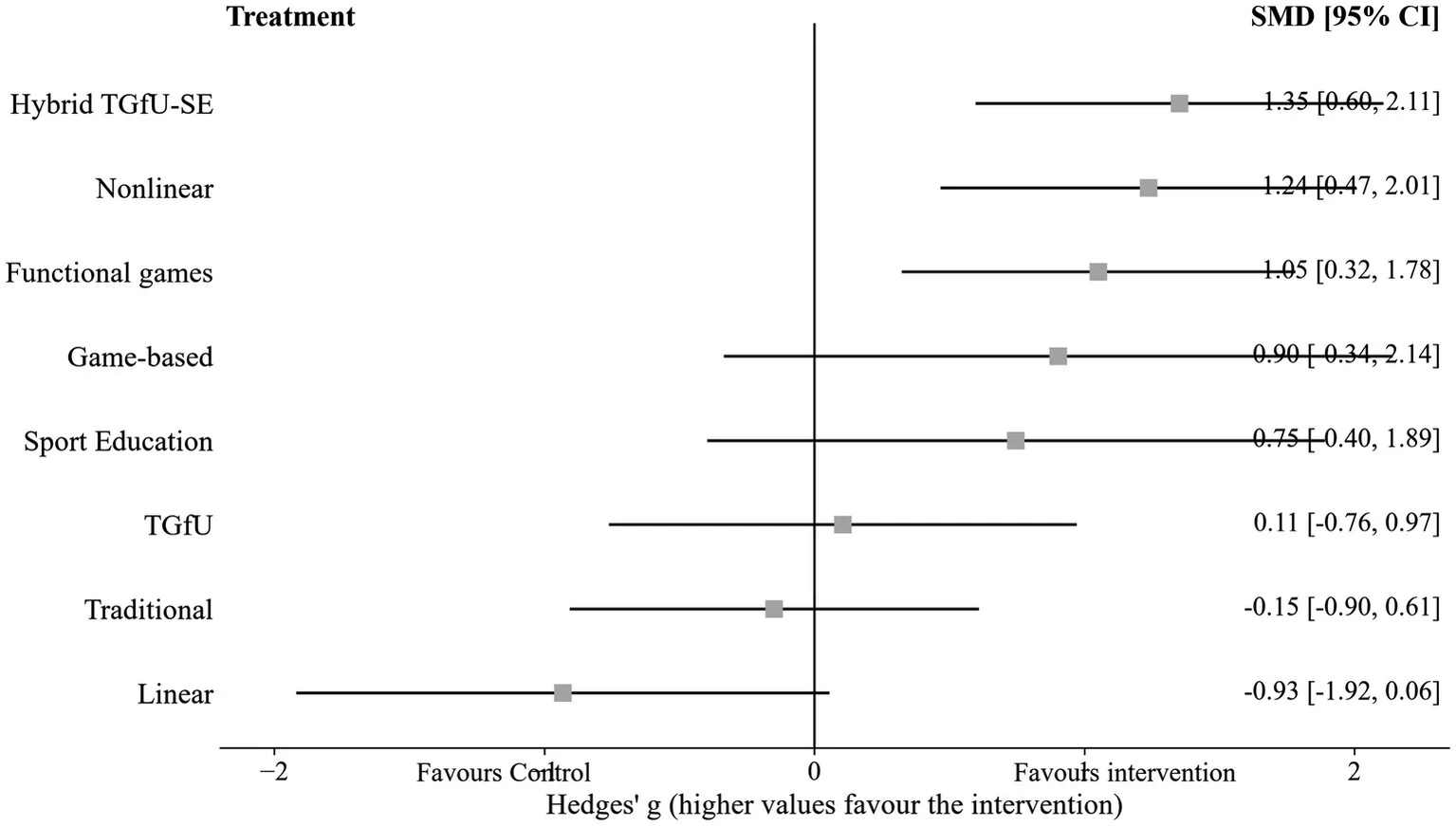
Forest plot of the 13-study under-18 primary network.

The *P*-score ordering for the primary network is shown in Figure 4. This ordering summarizes relative effect estimates and does not represent certainty of evidence.

**Figure 4:**
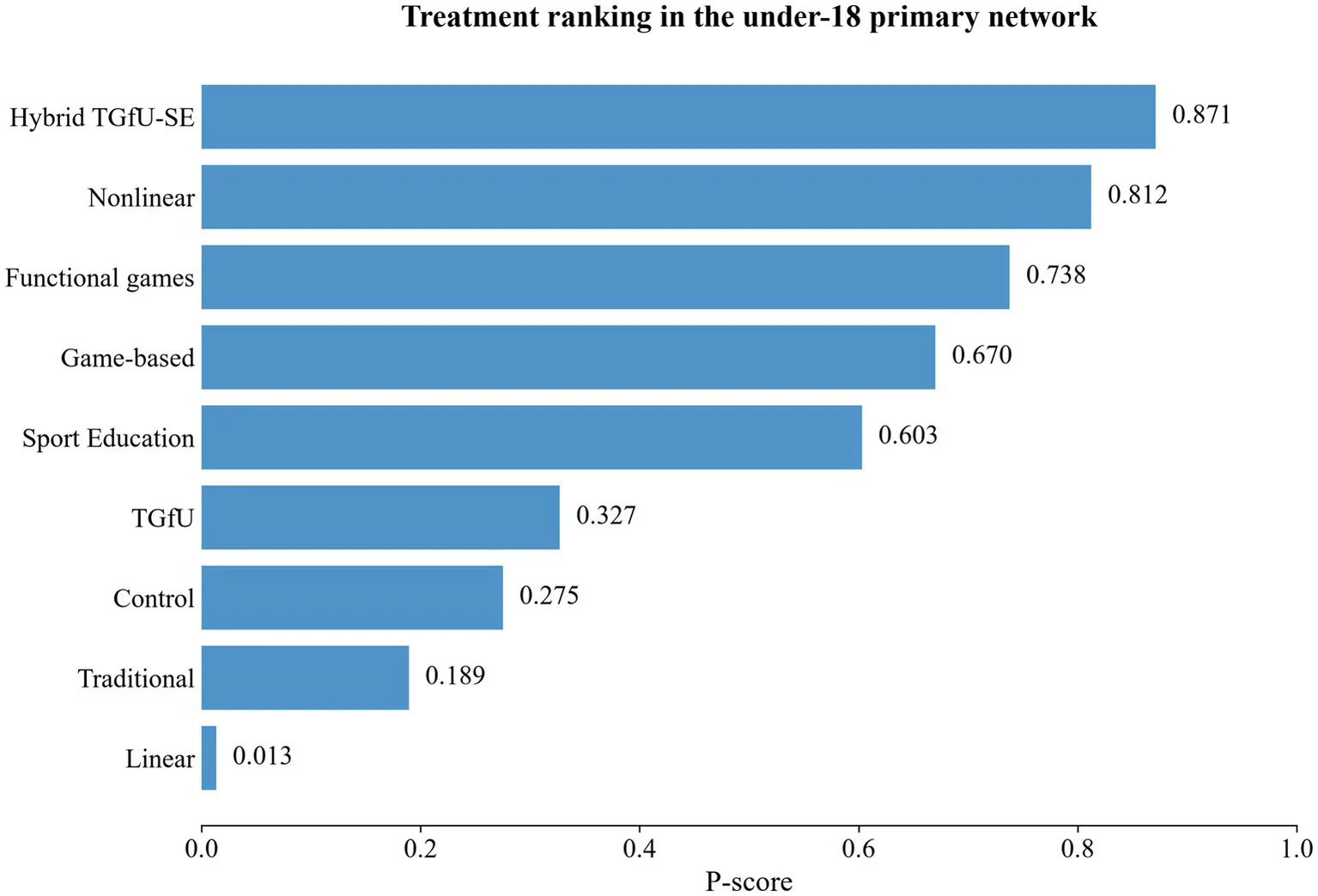
*P*-score ordering of relative effect estimates in the 13-study under-18 primary network. *P*-scores do not represent certainty of evidence.

### Sensitivity and outcome-domain analyses

To evaluate the influence of Akil (39) and Pugliese (44), the broad eligible network was refitted after excluding each study separately and then jointly (Table 5). In the 19-study broad network, Cooperative produced the largest estimated effect and the highest *P*-score (*P*-score = 0.994; SMD = 3.855, 95% CI 2.141 to 5.568), but this node was defined solely by Akil (39) and therefore did not constitute replicated evidence.

**Table 5:** Outcome-definition, age, variance-source, and study-influence sensitivity analyses.

Analysis	Studies	Leading node	*P*-score	SMD vs. Control (95% CI)	Tau-squared / I-squared
Under-18 primary network	13	Hybrid TGfU-SE	0.871	1.353 (0.598 to 2.107)	0.1531/65.67%
Broad eligible network	19	Cooperative	0.994	3.855 (2.141 to 5.568)	0.4238/80.89%
Under-18 broad network	17	Cooperative	0.995	3.800 (2.125 to 5.475)	0.3983/81.31%
Physical-motor outcomes only, all ages	16	Hybrid TGfU-SE	0.847	1.505 (0.548 to 2.461)	0.2948/72.64%
Primary excluding Orangi (47)	12	Hybrid TGfU-SE	0.941	1.526 (1.102 to 1.951)	0.0000/0.00%
Primary excluding Sgro (48)	12	Hybrid TGfU-SE	0.858	1.353 (0.495 to 2.211)	0.2165/72.53%
Broad excluding Akil (39)	18	Hybrid TGfU-SE	0.786	1.772 (0.717 to 2.827)	0.4238/80.89%
Broad excluding Pugliese (44)	18	Cooperative	0.992	3.889 (2.028 to 5.750)	0.4880/82.32%
Broad excluding both studies	17	Hybrid TGfU-SE	0.776	1.789 (0.644 to 2.934)	0.4880/82.32%

After Akil (39) was removed, the Cooperative node disappeared and Hybrid TGfU-SE had the highest *P*-score (0.786; SMD = 1.772, 95% CI 0.717 to 2.827). Because Cooperative was a leaf node, its removal left the estimates among the remaining connected treatments numerically unchanged. Excluding Pugliese (44) alone left Cooperative with the highest *P*-score (0.992) and produced only small changes in the remaining point estimates; for example, the Nonlinear estimate changed from 1.417 to 1.393. After both studies were excluded, Hybrid TGfU-SE had the highest *P*-score (0.776), followed by Game-based (0.712) and Functional games (0.706).

Joint exclusion did not reduce heterogeneity (tau-squared = 0.4880; *I*-squared = 82.32%, compared with tau-squared = 0.4238 and *I*-squared = 80.89% in the broad network). These exclusions therefore changed the conceptual composition and interpretation of the ranking more than they explained statistical heterogeneity. The *P*-score shifts should not be interpreted as corresponding changes in certainty of evidence.

To isolate the effect of outcome definition from population and setting, the broad network was refitted after excluding only Septiyanto (43), Akil (39), and Pugliese (44). The resulting 16-study physical-motor outcome network retained Práxedes (45), Yang Q (42), and Espoz-Lazo (46). Relative to the 19-study broad network, tau-squared decreased from 0.4238 to 0.2948 and I-squared from 80.89 to 72.64%; the between-design component was no longer statistically significant (*Q* = 2.69, df = 2, *p* = 0.260). Cooperative disappeared because Akil (39) was its sole study, and Hybrid TGfU-SE had the highest *P*-score (0.847; SMD = 1.505, 95% CI 0.548 to 2.461). The same three nodes led the 16-study and 13-study networks, although Functional games and Nonlinear exchanged second and third positions. This pattern indicates that both outcome definition and age eligibility contributed to ranking instability.

Age classification identified eight child/preadolescent studies (≤12 years), nine adolescent studies (13–17 years), and two young-adult studies (≥18 years). Removing Yang Q (42), which enrolled university men with mean ages of 21.05 and 21.00 years, from the former 14-study physical-motor network produced the 13-study under-18 primary network. Hybrid TGfU-SE remained first (*P*-score = 0.871), while Nonlinear moved to second and Functional games to third. Tau-squared decreased from 0.2846 to 0.1531 and *I*-squared from 74.45 to 65.67%; however, the between-design component became statistically significant (*Q* = 12.91, df = 2, *p* = 0.0016). The age restriction therefore improved population alignment and reduced heterogeneity but did not resolve inconsistency.

The child/preadolescent and adolescent broad-outcome subgroup networks each formed one connected component with one independent cycle. In the child/preadolescent subgroup, Cooperative had the highest *P*-score and estimated effect (*P*-score≈1.000; SMD = 3.297, 95% CI 2.440 to 4.153), but this estimate remained entirely dependent on the Akil (39) visual-motor integration study. In the adolescent subgroup, Functional games had the highest P-score (0.830; SMD = 2.568, 95% CI 1.277 to 3.859), followed by Game-based and Sport Education; between-design inconsistency remained significant (*p* = 0.0046). The young-adult subgroup contained only two studies and one edge. When mixed/cognitive-perceptual outcomes were removed, both the child/preadolescent and adolescent physical-outcome subgroup networks became connected trees without a closed loop, so inconsistency could not be assessed and separate all-treatment rankings were not treated as robust.

The source-level variance audit showed that 12 of 13 primary-network effects (92.31%) and 17 of 19 broad-network effects (89.47%) were calculated from reported arm-level sample sizes, means, and standard deviations. Orangi (47) was the only primary-network study for which the selected effect was reconstructed from reported standardized effects: competence-stratified Cohen’s *d* values of 4.13 and 2.14 with n = 16 per arm were converted to Hedges’ *g* and pooled. In the broad sensitivity network, Pugliese (44) was the only additional reconstructed effect; its reported adjusted *d* = 0.88 and group sizes of 98 and 67 were used. Descriptive arm statistics were reported by Pugliese (44), but the article’s adjusted effect was retained for this sensitivity outcome. No fixed or common standard error was imputed.

A prespecified leave-one-study-out analysis assessed the influence of Orangi (47) (Table 5). Excluding this study reduced tau-squared from 0.1531 to approximately zero and *I*-squared from 65.67 to 0%; total heterogeneity was no longer statistically significant (*Q* = 3.04, df = 4, *p* = 0.551), and the between-design component was also non-significant (*Q* = 1.39, df = 1, *p* = 0.239). Hybrid TGfU-SE remained first (*P*-score = 0.941; SMD = 1.526, 95% CI 1.102 to 1.951), and Functional games moved from third to second (*P*-score = 0.795; SMD = 1.308, 95% CI 0.901 to 1.716). Game-based moved to third, while Nonlinear moved from second to fifth. The leading node was stable, but the finer ordering, heterogeneity, and inconsistency estimates were sensitive to the reconstructed Orangi effect.

Because Sgro (48) was retained despite a serious ROBINS-I judgment, we repeated the under-18 primary NMA without that study. The exclusion left all nine treatment ranks unchanged: Hybrid TGfU-SE remained first (*P*-score = 0.858; SMD = 1.353, 95% CI 0.495 to 2.211), followed by Nonlinear and Functional games. Heterogeneity increased from tau-squared = 0.1531 and I-squared = 65.67% to tau-squared = 0.2165 and I-squared = 72.53%, while the between-design component remained significant (*Q* = 13.66, df = 2, *p* = 0.0011). This analysis indicates that Sgro (48) did not drive the treatment ordering or inconsistency signal, although its serious risk of bias continues to limit confidence in the evidence.

Exploratory outcome and age strata were then audited for network estimability (Table 6). Within the under-18 primary eligibility gate, general/fundamental motor skills included four studies across six nodes split into two components, and sport-specific physical performance included nine studies across nine nodes split into two components. The three mixed/cognitive-perceptual studies in the broad network also formed two components across five nodes. Separate all-treatment outcome-subgroup rankings were therefore not reported because all three outcome-domain networks were disconnected. The age-stratified broad networks and their corresponding physical-outcome networks are reported in Table 6 and interpreted according to connectivity and closed-loop availability. Práxedes (45) and Espoz-Lazo (46) were retained in the all-age 16-study physical-outcome sensitivity network but excluded from the primary sport-specific subgroup audit because their club and university settings did not meet the primary eligibility gate. Yang Q (42) was retained in that all-age sensitivity network but excluded from the under-18 primary outcome-domain audit because it enrolled university men.

**Table 6:** Outcome-domain and age-group subgroup network feasibility.

Subgroup	Studies	Nodes	Components	Closed loops	Inferential status
General/fundamental motor skill (under-18 primary)	4	6	2	0	Disconnected; one all-treatment NMA is not estimable
Sport-specific physical performance (under-18 primary)	9	9	2	0	Disconnected; one all-treatment NMA is not estimable
Mixed/cognitive-perceptual (broad only)	3	5	2	0	Disconnected; one all-treatment NMA is not estimable
Child/preadolescent, broad outcomes	8	7	1	1	Connected with one independent cycle; exploratory ranking estimable
Adolescent, broad outcomes	9	7	1	1	Connected with one independent cycle; exploratory ranking estimable
Young adult, broad outcomes	2	2	1	0	Connected tree; inconsistency cannot be assessed
Child/preadolescent, physical outcomes	6	6	1	0	Connected tree; inconsistency cannot be assessed
Adolescent, physical outcomes	8	7	1	0	Connected tree; inconsistency cannot be assessed

### Heterogeneity, inconsistency, and small-study effects

The 13-study under-18 primary network retained substantial heterogeneity (tau-squared = 0.1531; I-squared = 65.67%). The between-design component of the design-by-treatment decomposition was statistically significant (*Q* = 12.91, df = 2, *p* = 0.0016), as it was in the broad sensitivity network (*Q* = 25.77, df = 4, *p* < 0.001). Restriction to participants younger than 18 years in school-based physical motor and sport-performance settings reduced heterogeneity and aligned the analysis with the stated population, but it did not establish transitivity or eliminate inconsistency.

Node splitting did not identify a statistically significant local comparison in the 19-study broad network (0 of 10 comparisons); the TGfU-Control split was closest to the threshold (*p* = 0.054). In the 13-study under-18 primary network, four of eight local comparisons were statistically significant (*p* = 0.039): Nonlinear-Control, Linear-Nonlinear, Linear-TGfU, and TGfU-Traditional. These linked discrepancies arose within the same sparse loop and should not be read as four independent failures. Table 7 reports the direct, indirect, and difference estimates for the primary network. The local findings reinforce the need to interpret *P*-scores as conditional rather than definitive rankings.

**Table 7:** Local node-splitting results for the 13-study under-18 primary network.

Comparison	Direct SMD (95% CI)	Indirect SMD (95% CI)	Difference (95% CI)	*p*
Functional games vs. Control	1.140 (0.227 to 2.052)	0.900 (−0.313 to 2.112)	0.240 (−1.277 to 1.757)	0.757
Hybrid TGfU-SE vs. Control	1.728 (0.790 to 2.667)	0.665 (−0.604 to 1.935)	1.063 (−0.516 to 2.642)	0.187
Nonlinear vs. Control	0.852 (0.000 to 1.705)	2.936 (1.147 to 4.725)	−2.084 (−4.065 to −0.102)	0.039
Functional games vs. Traditional	1.159 (0.528 to 1.790)	1.399 (0.019 to 2.779)	−0.240 (−1.757 to 1.277)	0.757
Hybrid TGfU-SE vs. Traditional	1.193 (0.345 to 2.042)	2.256 (0.925 to 3.588)	−1.063 (−2.642 to 0.516)	0.187
Linear vs. Nonlinear	−2.741 (−3.780 to −1.703)	−0.658 (−2.345 to 1.030)	−2.084 (−4.065 to −0.102)	0.039
Linear vs. TGfU	−0.447 (−1.502 to 0.608)	−2.530 (−4.208 to −0.853)	2.084 (0.102 to 4.065)	0.039
TGfU vs. Traditional	0.420 (−0.139 to 0.979)	−1.664 (−3.565 to 0.237)	2.084 (0.102 to 4.065)	0.039

Deletion diagnostics indicated that the mixed cognitive-psychomotor comparison reported by Septiyanto (43) was the main contributor to the broad-network inconsistency. Removing this study reduced the between-design statistic from *Q* = 25.77 (df = 4, *p* < 0.001) to *Q* = 2.70 (df = 3, *p* = 0.441). By contrast, removing Pugliese (44) alone left significant inconsistency (*Q* = 24.35, df = 3, *p* < 0.001), while removing Akil (39) produced no change because Cooperative was a leaf node. Septiyanto (43) supplied the sole direct TGfU-Control comparison. This edge was contrasted with indirect routes through Linear and Nonlinear, through Traditional and Nonlinear, and through Traditional followed by Hybrid TGfU-SE or Functional games. The significant global test was therefore associated mainly with this edge across several overlapping loops, although no individual node split crossed the prespecified 0.05 threshold.

The significant between-design test and the four linked local splits show that inconsistency remains unresolved; few loops informed the assessment, and the local signals arose within the same sparse loop. Residual heterogeneity may reflect differences in age, sport content, intervention duration, implementation, and motor-performance instruments. The comparison-adjusted funnel plot was therefore used descriptively rather than as a definitive test of publication bias.

The comparison-adjusted funnel plot for the primary network is shown in Figure 5.

**Figure 5:**
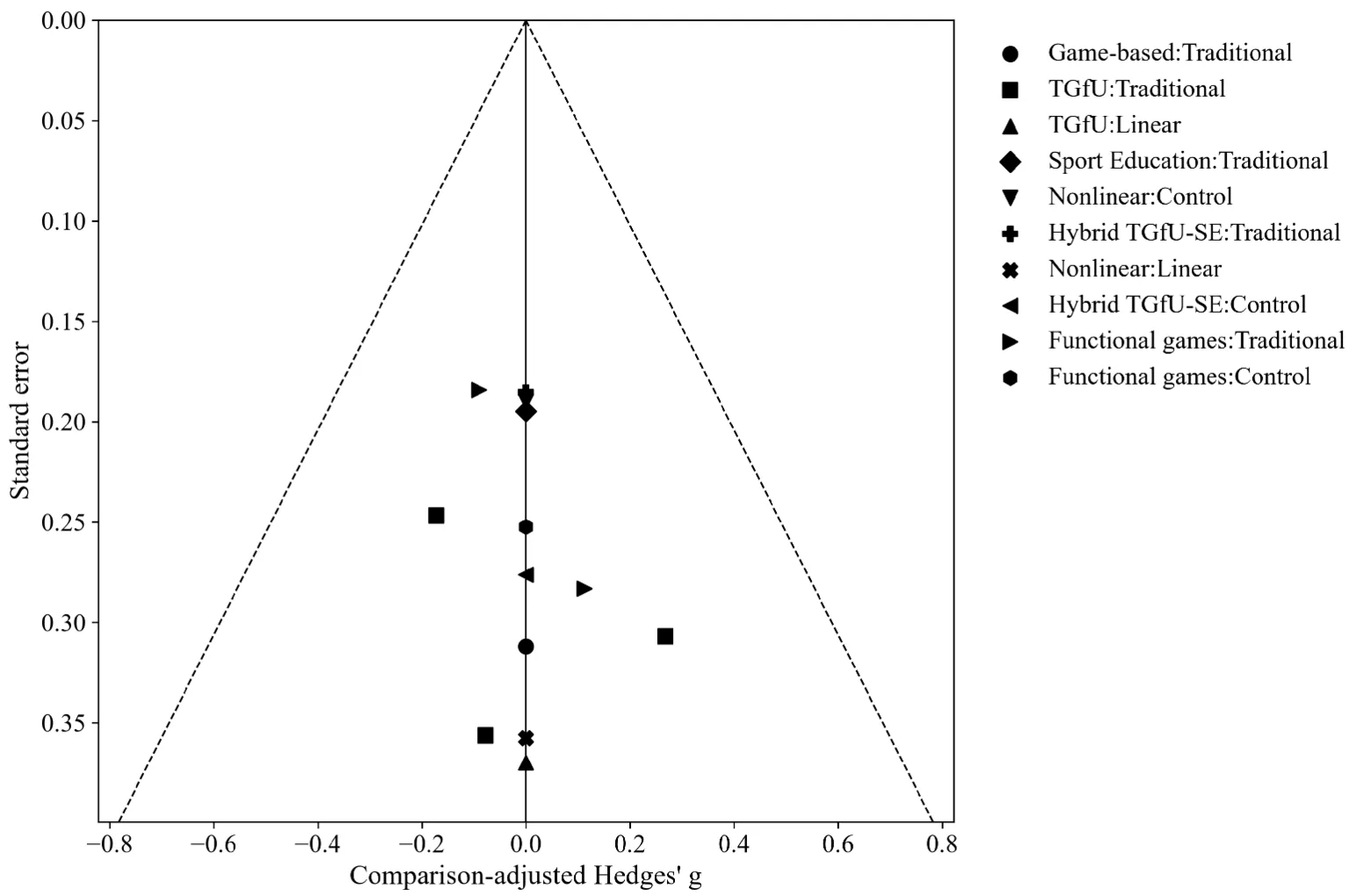
Comparison-adjusted funnel plot for the 13-study under-18 primary network.

## Discussion

### Main findings

The 13-study under-18 primary network did not support the earlier conclusion that all active teaching nodes outperformed Control. Hybrid TGfU-SE had the highest *P*-score and estimated effect, with Nonlinear and Functional games next in the *P*-score ordering; all three had positive confidence intervals relative to Control. This ordering describes relative effect estimates rather than a hierarchy of evidence certainty. Other nodes had imprecise estimates, so their ordering should not be interpreted as evidence of superiority or inferiority.

Effect estimates and evidentiary certainty are distinct. In the primary network, TGfU appeared in four studies and Functional games in three; Hybrid TGfU-SE and Nonlinear each appeared in two, whereas Game-based and Sport Education each relied on one. These node counts indicate replication within the network, not high certainty. No formal GRADE or CINeMA assessment was conducted, and P-scores should not be interpreted as certainty ratings. The broad sensitivity analysis further showed that Cooperative’s highest *P*-score depended entirely on the single Akil (39) VMI comparison. Significant inconsistency, wide intervals, and risk of bias limit confidence even for nodes supported by multiple studies.

Traditional instruction was approximately null relative to Control in the primary network. This finding does not establish that organized teacher-centered instruction is ineffective, but it does invalidate the earlier claim that Traditional clearly outperformed Control. Differences among comparator definitions and implementation quality remain plausible effect modifiers.

In terms of the literature on model-based teaching practices, which asserts that high-quality teaching is defined by the harmony between instructional design, educational outcomes, and the environment of the classroom rather than by one universal model, the readings can be considered quite supportive (10). Moreover, the readings are consistent with the results of umbrella reviews, according to which the most compelling benefit of model-based practices lies precisely in their design rather than the ranking order of individual models (16). This network emphasizes the fact that claims based on repetitive evidence patterns can be viewed as more valid than any higher-ranking evidence.

### Interpretation

Although the primary estimates for Game-based approaches and TGfU were imprecise, their pedagogical mechanisms remain theoretically plausible. Both approaches embed learning in representative game situations, encourage perception-action coupling, and often integrate decision-making with movement execution (13). These features may help explain why skills learned in context can transfer more readily than skills developed through isolated drills. Nonlinear approaches also fit established motor-learning theory emphasizing variation, exploration, and adaptive movement solutions (14, 24). Cooperative learning likewise remains theoretically attractive because peer interaction, shared responsibility, and verbalization can deepen engagement and reflection (10, 25), but Cooperative was absent from the primary network and appeared only in the broad sensitivity analysis.

Akil (39) was excluded from the primary network because visual-motor integration lies at the boundary of cognitive-perceptual integration and motor performance rather than directly measuring physical motor execution. In the broad sensitivity network, it was the sole study defining Cooperative and generated that node’s extreme rank. Its exclusion removed the Cooperative node rather than changing estimates among the remaining connected treatments. A slightly less obvious problem is associated with other studies that employed games performance, tactical-technical, balance, and cognitive-motor measures of motor skills that, while closely related to actual motor performance, are not fully interchangeable with TGMD tests (20). Outcome sensitivity is likely one of the factors responsible for the necessity to treat potentially promising game-based and/or cooperative approaches with caution (26, 27).

Outcome definition accounted for part of the instability observed in the broad network. Akil (39) was excluded from the primary network because visual-motor integration lies at the boundary of cognitive-perceptual integration and motor performance rather than directly measuring physical motor execution. In the nested 16-study physical-motor outcome network, excluding Septiyanto (43), Akil (39), and Pugliese (44) reduced tau-squared from 0.4238 to 0.2948 and I-squared from 80.89 to 72.64%; the between-design component changed from *p* < 0.001 to *p* = 0.260. Cooperative disappeared, and Hybrid TGfU-SE remained the leading node. However, the second and third positions differed from the 13-study under-18 primary analysis, indicating that age eligibility as well as outcome definition affected the ranking. Physical outcomes also continued to include general motor tests and sport-specific performance measures that are not fully interchangeable with TGMD-based assessments (20); the reduction should therefore be interpreted as partial rather than complete evidence about the source of heterogeneity (26, 27).

The local inconsistency analysis further clarifies why the rankings were unstable. In the broad network, none of the 10 node-splitting tests was individually significant, although the TGfU-Control split approached the threshold (*p* = 0.054). In the 13-study under-18 primary network, four linked comparisons within the same sparse loop had *p* = 0.039. Removing Orangi (47) eliminated this signal and reduced heterogeneity to approximately zero, whereas removing Sgro (48) did not resolve the between-design inconsistency. This pattern indicates that the apparent hierarchy depends on a small number of studies connecting Linear, Nonlinear, TGfU, Traditional, and Control. It supports conditional interpretation of the primary-network ranking; neither a significant global test nor several linked local splits identifies a single universally invalid teaching comparison.

Sport Education and Cooperative learning may also be especially sensitive to implementation quality. Both models rely on lesson structures that ask teachers to manage roles, peer interaction, accountability, and progression across time rather than simply deliver isolated drills (28). When implemented well, these features may deepen engagement and create rich learning environments; when implementation is partial or the unit is too short, the same models may not realize their theoretical advantages (29, 30). Recent syntheses of Sport Education and hybrid pedagogies likewise suggest that implementation conditions and participant characteristics can shape observed effects (31). This may help explain why nodes with attractive conceptual foundations can still appear statistically unstable in a sparse network (10).

### Methodological limitations

Several limitations remain. First, heterogeneity was substantial in the 13-study under-18 primary network (tau-squared = 0.1531; I-squared = 65.67%), and the between-design inconsistency component was significant (*p* = 0.0016). Four linked node splits within the same sparse loop had *p* = 0.039. Second, restriction to participants younger than 18 years and to physical motor execution or sport-specific performance improved population and outcome comparability but cannot prove transitivity; age, sport, duration, and implementation still varied. Third, Game-based and Sport Education were each informed by a single primary-network study, making their ranks imprecise. Fourth, 12 of 13 primary-network effects and 17 of 19 broad-network effects used reported arm-level sample sizes, means, and standard deviations. Orangi (47) in the primary network and Pugliese (44) in the broad sensitivity network used reported standardized effects and actual group sizes; no common or fixed standard error was imputed. Excluding Orangi reduced tau-squared from 0.1531 to approximately zero and I-squared from 65.67 to 0%, while Nonlinear moved from second to fifth. This finding identifies the reconstructed Orangi effect as a major contributor to residual heterogeneity and inconsistency and shows that fine-grained ordering remains sensitive to it. Established conversion principles were followed for these calculations (23). Fifth, the retrospective risk-of-bias appraisal suggested that none of the randomized comparisons could be considered unequivocally low risk and that all non-randomized comparisons were at serious risk, primarily because of confounding, non-random allocation, and incomplete reporting (21, 22). Excluding Sgro (48) left all nine treatment ranks unchanged but increased I-squared to 72.53% and left between-design inconsistency significant, so the study was retained because it met the primary eligibility gate and supplied extractable arm-level data; nevertheless, its serious ROBINS-I judgment remains a reason to interpret evidence from the TGfU-Traditional comparison cautiously.

The primary analysis applied the stricter population, setting, outcome, and under-18 age gate, while six connected but conceptually or contextually outlying studies were retained only in the broad sensitivity network. This restriction reduced tau-squared from 0.4238 to 0.1531 and I-squared from 80.89 to 65.67%, but the between-design inconsistency test remained significant (*p* = 0.0016). The child/preadolescent and adolescent broad-outcome networks were connected, yet their leading nodes differed and the child result was driven by the single Akil (39) VMI study. Once mixed/cognitive-perceptual outcomes were removed, both age-specific physical-outcome networks were trees without a closed loop. These findings indicate that age and outcome definition both contributed to instability but could not be disentangled cleanly in the sparse evidence structure. Replicating Cooperative, Game-based, and Sport Education using age-appropriate motor skill tests is necessary before their effect estimates can be generalized. Future studies should report complete arm-level means, standard deviations, sample sizes, and uncertainty for adjusted effects so that sampling variances can be calculated directly rather than reconstructed from standardized effects (23).

Another priority is greater conceptual standardization in how physical education studies define motor-skill outcomes. A network built from locomotor tests, sport-specific technique tests, game-performance instruments, and broader psychomotor composites can be informative, but only if researchers describe clearly what construct each instrument is intended to represent. Better reporting of intervention fidelity, teacher preparation, and lesson dosage would also help explain why nominally similar models sometimes produce substantially different effects (10, 28, 32).

While limited by certain constraints, the review pushes the interpretation further than mere juxtapositions of study findings. It specifies clearly where the evidence is abundant, where it is lacking, and why some strong-looking rankings need to be discounted. This approach is especially useful in the field of physical education, where support for novel teaching approaches often precedes solid comparative evidence. In this way, the current paper not only provides a ranking of effects but also offers a call for more rigorous interpretation of meta-analytical data in the educational sphere (33).

### Implications for practice

Within the constraints of the primary network, Hybrid TGfU-SE, Functional games, and Nonlinear showed positive estimates relative to Control with confidence intervals excluding zero. Estimates for Game-based, Sport Education, TGfU, Traditional, and Linear remained imprecise. These rankings should therefore inform, rather than prescribe, curriculum decisions (15, 34, 35).

From a curriculum perspective, the findings support investment in teacher professional development around model-based practice rather than reliance on a single default teaching script (32). Schools may not need one universally best model; they need teachers who can implement structured, evidence-informed approaches with fidelity and adapt them to age, sport content, and available lesson time (10, 28). This is especially relevant where physical education time is limited and each unit must accomplish both engagement and skill-development goals.

For schools and teacher-education programs, the implication is not that every lesson should be converted into the same model. Rather, curricula should build teachers’ capacity to select and implement structured models strategically. TGfU and broader Game-based approaches may be particularly suitable when the objective is to combine participation, tactical awareness, and motor performance in the same unit, whereas more technical or linear approaches may still play a supporting role when discrete skill refinement is needed (26, 36, 37). The same principle applies to hybrid or extended Sport Education designs, where context and enactment can shape the apparent payoff of the model (38). The main risk is not using the “wrong” named model once, but relying too heavily on low-structure instruction that provides too little purposeful practice (34). PETE programs could operationalize this capacity through supervised microteaching and lesson-design laboratories in which preservice teachers plan, teach, and revise short units using model-based approaches. Feedback could focus on implementation fidelity and on justified adaptations to task constraints, questioning, grouping, and lesson progression for different ages, skill levels, content, and time limits.

## Conclusion

This systematic review retained 21 studies, of which 13 contributed to the under-18 primary physical-motor network and 19 to the broad sensitivity network. In the primary analysis, Hybrid TGfU-SE had the highest *P*-score and estimated effect, followed by Nonlinear and Functional games. These values describe relative effect estimates; they are not certainty ratings. TGfU and Functional games appeared in four and three studies, respectively, while Hybrid TGfU-SE and Nonlinear appeared in two each. Game-based and Sport Education each relied on one primary-network study, and Cooperative was absent from the primary network; its leading broad-network estimate came solely from the Akil (39) VMI comparison. Estimates from these single-study nodes cannot be generalized.

The nested outcome-domain analysis showed that removing the three mixed/cognitive-perceptual studies reduced tau-squared from 0.4238 to 0.2948 and I-squared from 80.89 to 72.64%, while the between-design inconsistency test changed from *p* < 0.001 to *p* = 0.260. Applying the additional under-18 gate produced the 13-study primary network, in which Hybrid TGfU-SE had the highest *P*-score, followed by Nonlinear and Functional games. Heterogeneity decreased further, but significant global and local inconsistency, sparse nodes, age- and outcome-dependent effect ordering, and risk of bias prevent a definitive hierarchy.

The practical implication is not that one model is universally best, but that several structured approaches may improve motor performance when implemented well. Future studies should use comparable motor outcomes, report complete arm-level statistics, and replicate sparsely represented models before treatment rankings are used to guide policy or curriculum design.

## Data Availability

All data analyzed during this study are derived from published articles included in the systematic review and network meta-analysis. The datasets supporting the conclusions of this article are available from the corresponding authors upon reasonable request.
